# Reduced multisensory facilitation in adolescents and adults on the Autism Spectrum

**DOI:** 10.1038/s41598-019-48413-9

**Published:** 2019-08-19

**Authors:** Alexia Ostrolenk, Vanessa A. Bao, Laurent Mottron, Olivier Collignon, Armando Bertone

**Affiliations:** 1 Perceptual Neuroscience Lab for Autism and Development (PNLab), McGill University, Montreal, Canada; 2 School/Applied Child Psychology, Department of Education and Counselling Psychology, McGill University, Montreal, Canada; 3 University of Montreal Center of Excellence for Pervasive Developmental Disorders (CETEDUM), CIUSSS du Nord-de-l’Île de Montréal, Montreal, Canada; 4 Centre for Mind/Brain Science (CIMeC), University of Trento, Trento, Italy; 5 Institut de recherche en Psychologie (IPSY) et en Neuroscience (IoNS), Université de Louvain-la-Neuve, Ottignies-Louvain-la-Neuve, Belgium

**Keywords:** Perception, Sensory processing

## Abstract

Individuals with autism are reported to integrate information from visual and auditory channels in an idiosyncratic way. Multisensory integration (MSI) of simple, non-social stimuli (i.e., flashes and beeps) was evaluated in adolescents and adults with (n = 20) and without autism (n = 19) using a reaction time (RT) paradigm using audio, visual, and audiovisual stimuli. For each participant, the race model analysis compares the RTs on the audiovisual condition to a bound value computed from the unimodal RTs that reflects the effect of redundancy. If the actual audiovisual RTs are significantly faster than this bound, the race model is violated, indicating evidence of MSI. Our results show that the race model violation occurred only for the typically-developing (TD) group. While the TD group shows evidence of MSI, the autism group does not. These results suggest that multisensory integration of simple information, void of social content or complexity, is altered in autism. Individuals with autism may not benefit from the advantage conferred by multisensory stimulation to the same extent as TD individuals. Altered MSI for simple, non-social information may have cascading effects on more complex perceptual processes related to language and behaviour in autism.

## Introduction

Individuals with autism often avoid certain sensory stimuli (e.g., withdrawing from specific noises like the sound of a vacuum cleaner, avoiding certain textures or smells) and/or seek out sensory experiences through stimulatory behaviours (e.g., peering, echoing, tapping surfaces)^1,2^. The prevalence of sensory issues in autism is thought to vary between 69 and 95%, which confirms that sensory abnormalities are a concern for the vast majority of individuals with autism^3^. Furthermore, sensory issues have been shown to occur across development in autism^3,4^, as well as across sensory modalities^1^. Atypical reactivity to sensory input is now included among the DSM-5 symptoms for Autism Spectrum Disorder^5^. Autism Spectrum Disorder will continue to be referred to as “autism” throughout the text.

In light of the significance of sensory processing abnormalities, it has been suggested that these may actually contribute to some of the core social and behavioural characteristics of autism^6,7^. If sensory processing was altered, there would be a subsequent effect on higher-order processes. For instance, disruption in basic visual or auditory processing may contribute to deficits found at the higher level, such as socio-communicative functioning^7^. In fact, studies have demonstrated a relationship between sensory processing issues and social responsiveness^8^, communicative impairments and maladaptive behaviours^9^, as well as behavioural/emotional problems^10,11^.

The study of unimodal integration (e.g., integrating multiple visual stimuli into a whole) can be helpful to better understand different unisensory experiences. However, multisensory integration (MSI) may be a more ecological construct in that it better reflects naturalistic sensory experiences given that most of the situations that are encountered involve stimulation of more than one sensory modality at a time^12,13^. Multisensory integration is the process by which information from multiple sensory modalities are integrated into a whole^14,15^. The main advantage of MSI is that it allows to process incoming information more quickly and effectively^15^. In fact, the advantage conferred by MSI, referred to as multisensory facilitation, goes beyond what would be expected due to the effect of sensory redundancy^16^.

Various cognitive theories have hypothesized that there may exist altered sensory integration in autism, and that this alteration may be at the root of many diagnostic features of autism^17–19^. Deficits in sensory integration would potentially lead to a disjointed, confusing, and overwhelming perception of the physical surroundings^20^. Sensory aversion and sensory seeking behaviours in autism, as well as social withdrawal, and communication difficulties may then be partially explained as an effort to cope with the overload of information.

As the wide-ranging implications of MSI alterations in autism have become more apparent, studies using a variety of different paradigms to evaluate this area of functioning have emerged. Some such approaches have included complex stimuli and task demands, like the ability to integrate audiovisual information to process emotional expressions^21^, the speech-in-noise paradigm^22,23^, and the McGurk effect task^24–30^. Other researchers have investigated the issue using more simple approaches using non-social stimuli, such as the flash-beep illusion task^31–34^, and visual search tasks^35,36^.

The simple reaction time (RT) task has been frequently used to investigate the ability to integrate basic auditory and visual information in clinical populations (e.g., Schizophrenia^37,38^; Developmental Dyslexia^39^; Parkinson’s Disease^40^) and non-clinical populations^41,42^. During the audiovisual version of the RT task, participants are exposed to 3 conditions: (1) visual only, (2) auditory only, and (3) audiovisual multisensory presentation. The expectation is that typical multisensory function would lead to a facilitatory effect (i.e., significantly faster reaction time) during the audiovisual condition relative to the two unimodal conditions.

Despite the fact that the RT paradigm is arguably the simplest and most straightforward approach to investigate multisensory facilitation, only one study to date has used it to evaluate MSI in children and adolescents (7–16 years old) with autism using simple, non-social audiovisual stimuli^43^. While the typically-developing comparison group showed evidence for multisensory facilitation, in autism, both the younger (ages 7–10) and older (ages 11–16) age groups did not. Given the need to evaluate MSI using simple, non-social stimuli in order to better conceptualize this area of functioning in autism, and the fact that this basic task has never been completed with an older autistic population, the current study aims to fill the gaps in the literature, and help to better define MSI functioning in autism.

## Methods

### Participants

This study included a total of 40 participants, of which 20 were individuals diagnosed with Autism Spectrum Disorder (referred to as the AS group) and 20 were typically developing (TD group, only 19 included in analyses). In each diagnostic group, 10 participants were adolescents (age range 13–17) and 10 were adults (age range 18–29). Participants for the AS group were recruited from the Clinique d’Évaluation des Troubles Envahissants du Développement (CETED) database, at the Rivière-des-Prairies Hospital in Montreal. The TD participants were recruited via the CETED as well as through McGill University. Participants in the AS group were diagnosed based on DSM-IV criteria as well as the Autism Diagnosis Interview-Revised^44^ and/or the Autism Diagnosis Observation Schedule^45^ conducted by trained clinicians. Exclusionary criteria for both groups were assessed via a semi-structured interview and included: diagnosis of schizophrenia, ADHD, epilepsy, history of seizures, head injury, current use of stimulant medication or psychoactive drugs, the use of a hearing aid, and cochlear implants. Inclusionary criteria included: normal or corrected-to-normal vision (as measured by a vision test in the laboratory before beginning testing), and normal hearing (as measured by an auditory acuity test in the laboratory before beginning testing). Furthermore, all participants had to meet the minimum requirement of obtaining a full-scale IQ above 70 using Wechsler intelligence measures (i.e., WISC-IV^46^, WAIS-IV^47^, or WASI-II^48^). Typically-developing participants were screened for personal or familial history of autism. All participants, and their parent or legal guardian in the case of minor participants, gave written, informed consent before participating. This study was approved by Rivière-des-Prairies hospital’s and McGill University’s ethic committees. See Table 1 for participant demographics.

**Table 1:** Participant Demographic Variables by Group. Only 19 TD participants were included because one TD participant was excluded from the analysis (see section on “Outlier rejection process”).

	AS (n = 20)	TD (n = 19)	*t*	*p*
Sex				
Male	16	17		
Female	4	2		
Chronological Age			−0.252	0.802
*M*	19.21	19.61		
SD	4.71	5.15		
Range	13–29	13–28		
Age groups				
Adolescents	10	9		
Adults	10	10		
Wechsler Full-Scale IQ			−1.189	0.242
*M*	102.95	107.79		
SD	13.71	11.55		
Range	79–120	86–125		

### Apparatus

The stimuli were designed and presented using VPixx™ software and a MACPRO G4 computer, using an 18-inch Viewsonic E90FB 0.25 CRT (1280 × 1024 pixels) screen with a refreshing rate of 80 Hz. The mean luminance of the monitor was set at 30.00 cd/m2 (u’ = 0.1912, v’ = 0.4456 in CIE color space) where minimum and maximum luminance levels were 0.5 and 59.5 cd/m2, respectively. Viewing distance was set at 57 cm from the eyes of the participants to the center of the screen. Auditory stimuli were administered via the DataPixx™ data acquisition box. The auditory stimuli were presented in dichotic listening at 65 db SPL, with Sennheiser HD280 headphones. Stability of auditory intensity and visual luminance levels was ensured using a sonometer Quest 1100 and a CS-100 Minolta Chromameter, used for luminance/color reading and monitor gamma-correction.

### Stimuli and procedure

Multisensory integration was assessed using a target detection task^49^. Participants were either presented with the auditory stimulus alone (A trials; beep), the visual stimulus alone (V trials; flash), or both stimuli at the same time for the bimodal trials (AV trials). Participants were instructed to use the index finger of their dominant hand to press a button on a response pad as quickly and accurately as possible when a visual and/or and auditory target was detected. Participants began each new trial by pressing the space bar on a keyboard. Their reaction time (RT), defined by the time elapsed between the onset of the stimulus and the response button press, was recorded and used to measure performance. Unimodal and bimodal trials were interspersed randomly with blank catch trials (i.e., no visual or auditory stimulus presented) in order to reduce the likelihood of anticipatory responses.

Each participant completed a total of 4 trial blocks of 64 trials each, for a total of 256 trials, with short breaks between trial blocks. The first 4 trials of every block were practice trials to ensure that participants understood the process; these trials were not included in the analyses. The remaining 60 trials were presented in random order and included 15 blank catch trials, 15 auditory-only trials, 15 visual-only trials and 15 bimodal trials (audiovisual). Each trial began with a fixation cross-presented in the center of the screen for 1500 ms. For the active trials, the presentation of the fixation cross was followed by a visual stimulus, an auditory stimulus, or the simultaneous presentation of both stimuli after a variable random time delay of either 500, 750, 1000, 1250 or 1500 ms. The auditory stimulus consisted of a 3500 Hz tone presented binaurally through noise-cancelling headphones for a duration of 12.5 ms. The visual stimulus was a white disk subtending 3° of visual angle presented for a duration of 12.5 ms on a black background. Both the auditory and visual stimuli were highly salient and significantly above perceptual threshold in each modality. Participants were told at the start of the task that their reaction time was being measured, and to answer as quickly and accurately as possible when perceiving a visual and/or auditory stimulus.

### Analysis

#### Outlier rejection process

The goal of data cleaning was to eliminate trials in which the participant was distracted^50^. Responses with a reaction time inferior to 100 ms or superior to 1500 ms were excluded from the analysis. Other similar studies have used more restrictive limits, such as a maximum reaction time of 1000 ms^51^ or responses less than 3 standard deviations above or below the mean of a particular condition^37^. However, some of the autistic participants in the current study had generally slow motor responses that did not reflect distraction from the task. The upper limit of the inclusion window was increased to take participants with overall slower response speeds into account. The following rule was used to exclude any participant who was not paying attention to the task for an extended length of time: if 5 trials on any modality in a block were either unanswered or over/under the outlier threshold, then the whole block was removed from analysis. If 2 blocks out of 4 were invalidated in this process, then the participant was excluded. The outlier rejection process led to the exclusion of one TD participant who failed to answer 11 visual trials in the last two blocks. For the other participants, a total of 6 trials (0.18% of trials) were excluded from the TD group data, and 31 (0.86% of trials) from the AS group data (either because the participant missed them, or because they fell out of the 100–1500 ms window). In total, 20 participants with autism and 19 TD participants were included in the analysis.

#### ANCOVA

The effect of two independent variables (i.e., diagnostic group and trial type) on reaction time (dependent variable), controlling for age (covariate) were assessed using a 2-way mixed-factorial ANCOVA [2 (AS vs. TD) × 3 (auditory trials vs. visual trials vs. audiovisual trials)]. Greenhouse-Geisser corrections were applied when the Mauchly’s test of sphericity was significant in order to correct for the heterogeneity of variance. Based on the Bonferroni correction for multiple comparisons, our critical p-value was set at *p* < 0.005.

#### Race model analysis

The data was analyzed using Matlab (The Mathworks, Inc.) and a program called RMItest, written by Jeff Miller and described in Ulrich *et al*.^52^. Given that the bimodal stimuli provide two cues rather than one, the effects of multiple, redundant stimulation cannot be distinguished from the effects of multisensory facilitation by comparing the raw RTs for unimodal and bimodal stimuli. Subsequently, the race model analysis was used to determine whether any effect of quicker reaction time of AV trials went above and beyond the effect of redundant stimulation to indicate true multisensory facilitation.

The race model predicts that the reaction time to multimodal stimuli will be equal to the RT of the fastest individual stimulus (i.e. the RT to an audiovisual stimulus should be equal to the fastest RT observed for unimodal stimulation)^53^. If, however, reaction time to detect multimodal stimuli is significantly faster than for a unimodal signal, the race model prediction is violated, and this facilitation can be attributable to multisensory integration. The coactivation model stipulates that neural activations for both stimuli are combined and result in shorter reaction times^52,53^.

In the race model analysis, cumulative density functions (CDFs) of the RT distributions are generated for every participant and each experimental condition (i.e., visual alone, auditory alone, and bimodal condition). The CDFs obtained from the two unimodal conditions are then summed in order to compute the race model prediction for each participant. This measure provides an estimate of the boundary at which the race model inequality is violated. Percentile points are then determined for every distribution of RT, including the estimated bound for each participant. In the current study, the race model inequality was evaluated at 10 different points of the RT distributions (the 5th, 15th, 25th… 95th percentile points). The use of 10 percentiles is a good trade-off between resolution in separating the RT distribution without creating excessive multiple comparison issues due to the number of quantiles used. In other words, for each participant, the percentiles are computed taking the 5%, 10%, etc. trials with the shortest RT. The percentile values are then aggregated across participants in each group (AS vs. TD) e.g.the 5% fastest RTs for each participant in a given group are averaged together to give the group’s 5^th^ percentile mean RT. For each percentile, the group’s mean RTs for the bimodal condition are compared to the bound using a one-tailed paired-sample *t*-test. A Bonferroni correction for multiple comparisons is used to reduce Type 1 error. If the race model inequality is violated at the 5^th^ percentile, it means that the 5% fastest RTs for each participant in the bimodal condition, when grouped, were significantly faster than the race model predicts, (i.e. shorter than the estimated race model inequality bound). If any percentile shows significantly faster RTs in the bimodal condition relative to the bound, it can be concluded that the race model cannot account for the redundancy gain observed in the bimodal condition, supporting the existence of a multisensory integrative process. This analysis was computed for each diagnostic group as a whole, and for adolescents and adults separately in each group. We also analyzed individual data. For each individual in each group, the Race Model Inequality analysis was computed, and the reaction time to the AV condition was compared to the bound for each quantile and for each participant. This allowed us to estimate the percentage of participants in each group who had faster RTs than what was predicted by the Race Model, suggesting the existence of multisensory facilitation at the individual level.

### Ethics approval and consent to participate

All procedures performed in studies involving human participants were in accordance with the ethical standards of the institutional and/or national research committee and with the 1964 Helsinki declaration and its later amendments or comparable ethical standards. Ethics approval was obtained by the McGill University REB (#282–1215) and the Comité d'éthique de la recherche du CIUSSS du Nord-de-l'Île-de-Montréal for Hopital Riviere des Prairies (Project #16-02 P).

## Results

### ANCOVA results

**Figure 1:**
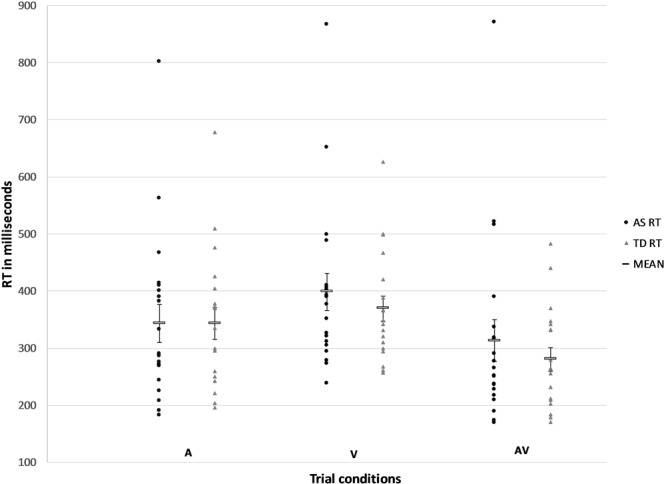
Mean reaction times (ms) for the AS and TD group across the trial conditions (audio (A), visual (V) and audiovisual (AV)). RTs for the AV conditions were significantly shorter than RTs for the V condition. Error bars represent the Standard Error of the Mean.

A 2-way mixed-factorial ANCOVA [2 (AS vs. TD) × 3 (auditory trials vs. visual trials vs. audiovisual trials)] was used to determine differences between groups and trial type, controlling for age (Fig. 1). There was no main effect of group, *F*(1, 36) = 0.106, *p* = 0.747, *η*_*p*_^2^ = 0.003, and no significant group x trial type interaction, *F*(1.36, 48.97) = 1.076, *p* = 0.326, *η*_*p*_^2^ = 0.029. Although pairwise comparisons indicate that there are significant differences conditions, after controlling for age, no main effect of condition was found, *F*(1.36, 48.97) = 1.056, *p* = 0.331, *η*_*p*_^2^ = 0.029.

### Race model analysis results

Prior to running the race model analysis, we computed the Redundancy Gain (RG) for each group. This allowed us to quantify the gain in RT that results from redundant stimulation as compared to unimodal stimulation. The RG is computed by subtracting the mean RT on bimodal trials from the mean RT of the fastest unimodal condition and dividing that by the mean RT for the fastest unimodal condition. The AS group demonstrated a redundancy gain (i.e. percentage decrease in RT on multimodal as compared to unimodal trials) of 6.2%, whereas the TD group had a redundancy gain of 15.8% (*t* = −1.80, *p* = 0.045). Although both groups experienced a gain in RT on the multimodal trials, the race model inequality analysis is necessary to distinguish between multisensory facilitation and the effects of redundant stimulation.

**Figure 2:**
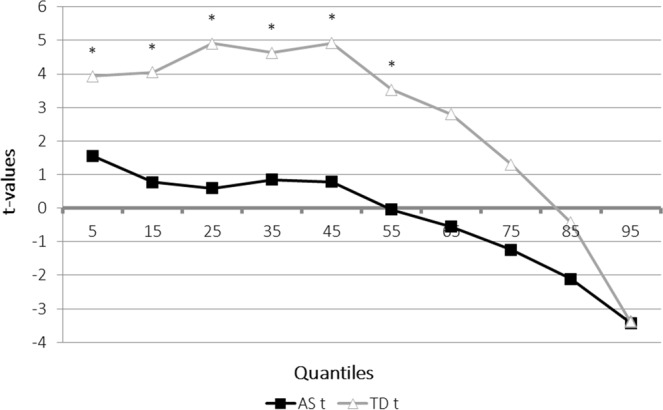
The graph represents the difference in milliseconds (Y axis) between the model prediction based on the auditory and visual conditions, and the RTs obtained in the audiovisual conditions for each group (AS and TD). Positive values represent RTs that were faster than the race model prediction. The difference between the bound (represented as 0 on the Y axis) and the RTs of the bimodal condition are computed for each percentile of the RT distribution (X axis). *indicates significant violation of the race model (*p* < 0.005).

The race model analysis showed different results according to the diagnostic groups (see Table 2 for detailed results). For the TD group, the bimodal stimuli significantly violated the race model assumption through the 55^th^ percentile of the reaction time distribution, suggesting that the redundancy gain could be explained by multisensory facilitation. However, in autism, there was no significant violation of the race model, showing no evidence for multisensory facilitation (Fig. 2).

**Table 2:** Race Model Inequality Analysis Results by Group.

Quantile	AS group				TD group			
	Mean RT (in ms) for AV trial	Bound	*t*-value	*p-*value	Mean RT (in ms) for AV trial	Bound	*t*-value	*p-*value
0.05	214.83	224.21	1.560	0.068	204.84	225.09	3.930	0.001*
0.15	242.64	248.82	0.767	0.227	224.78	248.91	4.046	0.001*
0.25	262.23	267.59	0.589	0.282	238.88	263.90	4.905	0.000*
0.35	276.83	283.86	0.847	0.204	251.58	276.87	4.628	0.000*
0.45	289.62	296.41	0.780	0.222	263.83	288.86	4.919	0.000*
0.55	307.87	307.45	−0.040	0.485	278.23	299.10	3.528	0.001*
0.65	324.80	317.84	−0.558	0.292	290.54	309.50	2.793	0.006
0.75	345.91	329.02	−1.240	0.230	309.22	320.46	1.303	0.105
0.85	388.12	338.96	−2.110	0.024	334.00	329.50	−0.431	0.336
0.95	460.15	350.44	−3.429	0.002	392.93	338.26	−3.399	0.002

*Indicates significant *p*-values for one-tailed, paired-samples *t*-test between mean RT for AV condition and the bound. Significance is set at *p* < 0.005.

**Figure 3:**
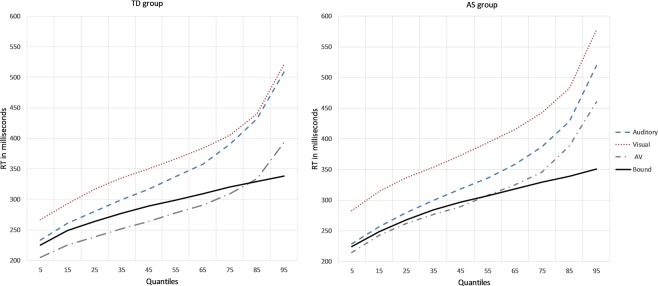
Reaction time distribution for unimodal and multimodal conditions compared to the Race Model bound TD ad AS groups.

Figure 3 further depicts the distribution of RTs for all three conditions across both groups as compared to the predicted bound. Similar results were found when the same analysis was run according to age group (i.e., adolescent and adult) in order to explore potential changes in MSI through development. In TD adults only, the same violation of the race model through the 55^th^ percentile was found. The violation was reduced to the 45^th^ percentile in TD adolescents. There was no significant violation of the race model for either adults or adolescents in the AS group. Although both groups showed similar reaction times in response to the three conditions, the race model analysis was able to identify a difference in the facilitation that can be specifically attributed to the bimodal nature of the trials.

**Figure 4:**
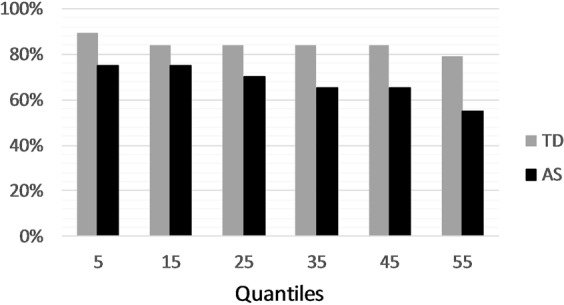
The percentage of participants in each group who show faster reaction times for the audiovisual condition than the bound predicted by the race model at each of the six quantiles considered. TD, participants with typical development; AS, autism spectrum participants.

At the individual level, 89% (N = 17) of the TD participants and 75% (N = 15) of the AS participants showed RTs in the audiovisual condition that were faster than the bound computed by the race model for at least one of the first six quantiles (i.e. the quantiles where violations of the race model were expected to occur). Figure 4 illustrates, for each of the first six quantiles, the percentage of participants in each group that had AV reaction times faster than the bound. We also calculated the number of quantiles where the RTs in the audiovisual condition were faster than the bound for each participant; a one-tailed *t*-test showed a significant difference across groups (*p* = 0.035), indicating that TD participants had faster AV RTs than the race model bound more frequently than AS participants. Detailed individual participants’ data can be found in the Supplementary Information.

## Discussion

Research investigating multisensory integration in autism has expanded, and a burgeoning body of literature exists specifically on the integration of auditory and visual information (e.g.^37,39,54^). However, one of the simplest and most straightforward approaches to investigating audiovisual MSI (i.e., the RT paradigm) has been underutilized to address the question of whether MSI is altered in autism. Only one study to date has used an RT task using simple, non-social stimuli (i.e., flashes and beeps) to investigate MSI in children with autism^43^. The current study aimed to expand on this work by using the RT task to evaluate MSI of audiovisual information in adolescents and adults with autism. Results indicate that only the TD group demonstrated multisensory facilitation, while the AS group showed no evidence of multisensory facilitation. The race model analysis determined that the TD group violated the race model assumption through the 55^th^ percentile (i.e., violation at any percentile is sufficient to provide evidence of multisensory facilitation). On the other hand, the AS group RTs did not violate the race model at any of the percentiles. These findings clearly indicate that TD individuals benefit significantly more (i.e., faster reaction times) from the concurrent presentation of multiple sensory stimuli, than individuals with autism. Overall, these results suggest that MSI is altered in autism for the most basic type of audio-visual information, using the simplest (RT) task.

These results are also consistent with many of the findings from tasks that have used socio-communicative stimuli and have supported a hypothesis of altered or reduced multisensory integration in autism^21,24,26,29,55^. In fact, the current results suggest that MSI is altered in autism regardless of the complexity of the task or stimuli used to assess this area of functioning. Despite the fact that these results are consistent with the findings of many other studies investigating MSI in autism, there remains inconsistency in the field. Specifically, some other studies have not shown differences between AS and TD individuals during tasks designed to assess MSI, or have found that differences may be stimulus- or task-dependent^25,29,34,36,56^. For instance, some research using the flash-beep illusion task has shown that individuals with autism demonstrate susceptibility to one or both of the flash-beep illusions^31,32,34^. The potential difference between those results and the results from RT tasks may lie in the use of different paradigms to assess MSI. In the RT task, MSI is measured within the context of multisensory facilitation (i.e., whether the presentation of two concurrent stimuli aids in the speed and efficiency with which sensory information is processed). On the other hand, with tasks like the flash-beep illusion or the McGurk effect, it is the automaticity and generalizability of MSI that can lead to faulty or illusory perception that is measured (i.e., whether perception is influenced by the tendency to integrate sensory information into one coherent percept). Individuals with autism may not be benefitting from multisensory facilitation, and instead rely more consistently on information from one modality at a time when performing speeded tasks, but may be susceptible to the same illusory perception as typically developing individuals when the addition of sensory stimuli serves to impede perception, rather than aid it.

This suggestion is consistent with the results of Collignon *et al*.^35^ in their evaluation of multisensory facilitation using a visual search paradigm. Both AS and TD participants performed two variations of a visual search task. In the simple visual search condition, participants had to find a visual target amongst distractor items, where the target as well as the distractors changed colour at random intervals. In the multisensory facilitation condition, participants were “aided” by a facilitatory sound that coincided with the target’s colour change. While the TD participants exhibited signs of multisensory facilitation (i.e., their search time was improved on the multisensory condition), the AS participants showed no such evidence of facilitation. Individuals with autism may be showing evidence of a local processing bias, which is consistent with accounts from the Weak Central Coherence theory^18^ as well as the Enhanced Perceptual Functioning hypothesis^19,56^. The predictive coding accounts of autism suggest that autistic individuals might give equal weight to all the cues they perceive and have a lower tolerance for slight spatiotemporal mismatch between cues, thus favouring the experience of distinct unimodal input and preventing optimal integration^57^.

Comparing the effect of multisensory facilitation across age groups using the race model yielded no further differences within the AS group, and only a slight difference within the TD group. Specifically, the race model was not violated at any of the percentiles neither for the adolescent nor the adult participants with autism, but was violated through the 45^th^ percentile for the TD adolescents and the 55^th^ percentile for the TD adults. Although the TD adolescents still exhibit the presence of multisensory facilitation, the effect is not as strong as it is for older participants. These results are in line with those of Brandwein *et al*.^43^ who demonstrated that the younger (7–10) and older (11–16) children with autism exhibited no significant race model violations, but that the younger TD participants exhibited fewer race model violations than the older TD children. Taking these results together, it may be the case that in typical development, the ability to integrate multiple sensory stimuli and perform with more speed and efficiency due to multisensory facilitation fully develops in later adolescence and adulthood. On the other hand, the results from both studies may support that individuals with autism continue to integrate sensory information atypically (i.e., benefit less from multisensory facilitation) throughout development. However, such a conclusion would need to be confirmed by longitudinal analyses of multisensory integration. Only by examining how this area of functioning progresses throughout development will the developmental trajectory of MSI be fully understood. Furthermore, as the population of individuals with autism continues to age, the MSI ability of older individuals should be taken into consideration to better understand the developmental trajectory of MSI in autism.

Although the current study addresses some of the gaps in the literature, it does present some limitations. For instance, the sample size is small and does limit the ability to generalize findings to the overall AS population or to look at age groups separately. In the future, recruiting larger samples to facilitate interpretation of reaction time data as well as performing age analyses will allow for better understanding of age effects on MSI in autism. In addition, although important with regards to understanding the mechanisms mediating MSI in autism, using simple, non-social stimuli is not as representative as the more complex, socially contingent information that define our natural environments.

Overall, the results of the current study help to support a growing body of literature that indicates that individuals with autism do not integrate sensory stimuli in an entirely typical fashion^43^. Most importantly, MSI in autism appears to be atypical using the RT paradigm which may be considered one of the most simple and elementary indices of MSI. This may suggest that the core features of autism have an early origin in the simple, non-social level of sensory processing^58^. If individuals with autism have difficulty integrating even simple information like flashes and beeps, then it follows that this altered processing has cascading effects on more complex perceptual processes like communication, social interaction, and interacting with the environment effectively^20,33,59,60^. Given the importance of this area of functioning, further research should continue to help disentangle the effects of multisensory integration difficulties from methodological differences across studies. In addition, future research focusing on bridging the gap between the neural mechanisms and the behavioural manifestations of MSI would allow for better understanding of the ways in which MSI differs in autism and the far-reaching consequences of such an alteration.

## Supplementary information

Supplementary Dataset 1

## Data Availability

The datasets generated during and/or analyzed during the current study are available from the corresponding author on reasonable request.
