# Supplementary material for: Reduced multisensory facilitation in adolescents and adults on the Autism Spectrum
Source: Sci Rep. 2019 Aug 19;9:11965. doi: 10.1038/s41598-019-48413-9 (PMC6700191; doi:10.1038/s41598-019-48413-9)
Supplement: Supplementary file 1 — Supplementary Dataset 1 [file 41598_2019_48413_MOESM1_ESM.pdf]

Supplementary Information

Reduced multisensory facilitation in adolescents and adults on the Autism Spectrum

[co] Alexia Ostrolenk[1,3], [co] Vanessa A. Bao[1,2], Laurent Mottron[3], Olivier Collignon[4,5], *Armando Bertone[1,2,3]

[co] These two authors contributed equally to this work.

Affiliations:

1. Perceptual Neuroscience Lab for Autism and Development, McGill University
2. School/Applied Child Psychology, Department of Education and Counselling Psychology, McGill University
3. University of Montreal Center of Excellence for Pervasive Developmental Disorders (CETEDUM)
4. Centre for Mind/Brain Science (CIMeC), University of Trento, Italy
5. Institut de recherche en Psychologie (IPSY) et en Neuroscience (IoNS), Université de Louvain-la-Neuve

*Correspondence to armando.bertone@mcgill.ca

Individual participants' mean RTs for audiovisual trials (Z, in ms) and race model bound computed at each quantile.

Autism Spectrum Group

**Participants**	**1**		**2**		**3**		**4**		**5**		**6**		**7**		**8**		**9**		**10**	
**Quantile**	**Z**	**Bound**	**Z**	**Bound**	**Z**	**Bound**	**Z**	**Bound**	**Z**	**Bound**	**Z**	**Bound**	**Z**	**Bound**	**Z**	**Bound**	**Z**	**Bound**	**Z**	**Bound**
**0.05**	234.03	258.00	351.90	293.89	207.23	215.88	164.71	189.22	460.43	422.49	218.93	222.90	332.33	409.33	164.48	176.00	191.15	188.30	152.95	172.78
**0.15**	258.41	281.65	404.70	330.08	227.64	246.72	176.15	199.54	604.00	496.10	246.02	252.25	396.46	422.13	176.82	200.00	222.23	211.88	178.60	194.91
**0.25**	279.55	305.00	457.65	370.64	237.28	258.64	187.16	209.71	693.46	572.91	258.07	304.02	436.34	434.22	199.63	218.44	231.85	223.92	187.52	202.98
**0.35**	297.98	315.80	483.82	399.70	246.81	266.22	192.63	211.58	759.38	662.93	269.26	320.96	457.18	465.14	211.82	234.41	246.11	242.12	199.29	212.46
**0.45**	318.67	322.11	506.25	413.94	258.85	281.35	199.51	213.62	788.80	695.77	289.33	347.21	475.05	480.43	223.71	242.72	252.55	258.03	211.45	224.30
**0.55**	341.66	328.46	519.02	437.34	281.41	293.59	211.46	218.61	877.65	729.10	305.00	360.22	510.25	492.24	235.21	246.71	269.28	270.50	237.53	234.37
**0.65**	351.85	338.48	539.06	452.44	293.37	304.79	219.00	222.34	939.76	741.70	325.47	380.57	551.24	504.24	251.00	258.07	279.40	281.42	254.20	240.13
**0.75**	387.75	347.02	574.70	462.89	307.34	315.03	231.41	225.08	981.28	760.54	355.87	398.28	574.53	518.05	278.83	263.73	296.28	287.53	272.22	246.59
**0.85**	416.40	360.09	621.60	470.66	328.73	328.58	246.74	230.62	1231.25	782.83	416.35	410.95	656.83	532.32	302.27	274.31	317.44	299.43	305.00	253.33
**0.95**	477.00	375.04	709.55	479.44	375.50	340.61	310.85	234.21	1439.55	806.89	451.65	426.37	821.00	552.47	392.95	282.97	341.49	316.13	375.35	259.44

**Participants**	**11**		**12**		**13**		**14**		**15**		**16**		**17**		**18**		**19**		**20**	
**Quantile**	**Z**	**Bound**	**Z**	**Bound**	**Z**	**Bound**	**Z**	**Bound**	**Z**	**Bound**	**Z**	**Bound**	**Z**	**Bound**	**Z**	**Bound**	**Z**	**Bound**	**Z**	**Bound**
**0.05**	185.35	200.00	164.43	173.05	134.95	140.68	269.85	293.77	141.84	154.95	219.56	210.62	145.19	140.59	228.55	266.47	164.09	176.33	164.65	178.93
**0.15**	200.57	219.90	181.85	199.92	152.10	164.12	293.01	318.75	158.85	167.14	231.70	236.73	152.68	150.83	246.79	299.50	168.12	188.17	176.11	196.09
**0.25**	210.63	235.30	187.78	211.80	152.97	164.80	316.06	328.54	164.40	176.41	246.11	260.73	161.30	152.83	265.87	324.03	188.34	197.23	182.54	199.70
**0.35**	221.70	244.27	199.20	224.76	164.01	169.18	342.54	335.89	167.70	186.54	250.27	275.30	164.39	164.64	275.61	335.57	199.05	199.97	187.88	209.81
**0.45**	223.98	251.52	211.24	237.19	164.72	175.01	375.20	342.34	176.36	188.94	259.16	292.38	164.90	175.64	293.02	347.43	200.20	211.33	199.51	226.87
**0.55**	235.30	269.08	222.13	246.59	171.44	176.54	398.05	351.46	178.05	200.10	273.17	305.74	170.43	181.85	297.37	352.59	211.11	216.87	211.78	237.03
**0.65**	235.82	277.15	246.47	254.61	175.80	183.42	410.50	363.27	188.49	206.79	293.93	321.58	176.18	187.25	327.41	364.29	211.80	223.59	225.24	250.78
**0.75**	247.96	303.50	268.34	265.33	176.86	197.17	422.93	375.02	200.00	221.16	328.50	328.92	180.32	187.99	352.91	375.71	233.77	231.60	246.47	269.24
**0.85**	272.57	310.91	293.66	281.19	187.46	199.58	451.55	383.75	221.16	225.94	348.00	340.48	187.65	199.18	406.79	378.35	246.67	234.89	304.36	281.82
**0.95**	305.90	321.21	434.00	291.13	205.35	203.39	527.75	395.79	276.40	237.12	422.46	351.70	211.50	201.82	486.95	393.48	266.29	246.34	371.43	293.27

	Participants	1		2		3		4		5		6		7		8		9		10	
	**Quantile**	**Z**	**Bound**	**Z**	**Bound**	**Z**	**Bound**	**Z**	**Bound**	**Z**	**Bound**	**Z**	**Bound**	**Z**	**Bound**	**Z**	**Bound**	**Z**	**Bound**	**Z**	**Bound**
Typically Developing Group	**0.05**	216.52	269.98	328.48	352.85	258.23	281.35	204.72	242.38	205.35	240.5	153	164.69	160.3	176	269.06	258.08	149.28	130.97	161.28	164.85
	**0.15**	222.58	293.45	358.92	386.35	283.84	319.72	229.4	259.73	224.57	258.12	164.42	176.44	168.98	187.72	293.02	284.46	164.14	152.36	173.58	183.86
	**0.25**	234.42	304.3	389.05	406.14	312.8	340.61	247	283.52	234.05	258.84	167.58	179.14	182.38	202.4	305.71	294.86	165.22	175.11	179.19	196.5
	**0.35**	246.2	310.51	412.45	434.56	340.94	367.55	256.2	294.53	234.86	264.23	176.08	187.01	195.89	213	326.08	305.86	172.49	184.89	187.6	200.04
	**0.45**	246.96	316.64	433.77	469.48	378.32	382.71	270.42	305.08	246.65	269.4	176.98	188.56	199.68	222.11	337.78	326.94	181.95	198.64	199.23	211.59
	**0.55**	258.08	325.07	469.01	504.02	434	395.47	282.47	311.07	258.66	277.26	187.81	199.35	209.81	222.78	345.52	332.37	187.75	207.64	206.75	217.75
	**0.65**	259.48	328.72	510.15	527.42	472.13	410.08	292.93	317.54	273.14	281.78	188.87	206.7	211.93	234.14	351.87	340.72	199.67	216.68	215.1	222.72
	**0.75**	267.74	340.1	566.9	554.22	527.83	433.13	297.54	321.83	287.4	291.27	199.28	215.5	222.16	239.86	387.08	347.51	214.13	222.6	232.93	231.8
	**0.85**	275.7	342.26	604.05	577.58	604.05	445.32	317.73	330.24	316.76	293.62	203.8	234.06	240.5	249.86	389.52	351.78	248.16	234.03	246.79	234.86
	**0.95**	308.9	350.52	770.13	598.71	709.6	457.04	364.51	340.26	340.66	297.39	222.12	240.99	281.8	259.48	410.51	359.72	311.39	237.62	304.95	246.09
	**Participants**	**11**		**12**		**13**		**14**		**15**		**16**		**17**		**18**		**19**			
	**Quantile**	**Z**	**Bound**	**Z**	**Bound**	**Z**	**Bound**	**Z**	**Bound**	**Z**	**Bound**	**Z**	**Bound**	**Z**	**Bound**	**Z**	**Bound**	**Z**	**Bound**		
	**0.05**	146.46	155.57	160.88	173.32	240.45	253.25	226.82	271.13	170.74	180	124.73	129.8	261.03	335.51	214.54	219.97	240.02	276.45		
	**0.15**	153.4	164.53	182.27	210.08	280.23	277.78	271.13	293.32	188.36	200	138.03	142.5	287.4	370.1	225.9	239.34	260.57	329.4		
	**0.25**	160.39	175.17	193.4	226.82	293.96	306.38	291.4	308.18	201.43	216.72	146.55	157.43	309.96	380.4	239.67	248.53	284.66	353		
	**0.35**	162.5	185.81	213.54	240.15	306.69	316.5	306.62	322.99	215.3	226.66	151.84	166.28	322.63	390.3	246.6	261.61	305.45	388.01		
	**0.45**	173.27	194.72	225.37	250.4	318.67	328.63	322.96	348.18	222.9	235.1	159.09	173.16	348	398.82	254.52	269.99	316.3	398.23		
	**0.55**	176.36	203.71	240	260.05	338.72	338.63	336.7	364.43	226.44	240.71	160.53	176.36	375.21	412.92	263.42	281.96	329.21	411.27		
	**0.65**	185.22	213.47	243.87	275.16	359.84	346.84	349.12	376.85	232.46	250.52	168.32	189.05	394.9	422.63	269.69	295.63	341.66	423.92		
	**0.75**	187	219	256.06	286.67	375.21	363.07	364.43	386.24	251.82	260	175.03	200.09	422.01	429.5	281.97	305.27	358.75	441.07		
	**0.85**	205.09	226.28	280.64	294.58	408.87	373.23	386.74	393.28	266.74	266.36	196.98	200.95	445.52	445.36	296.64	316.32	411.75	450.51		
	**0.95**	230.05	233.88	307	306.66	519.95	380.02	433.35	399.37	320.07	267.19	280.45	211.04	539.55	457.09	334.3	324.92	476.4	458.98		
